# Methodological Considerations in Meta-Analyses Examining Irregular Dietary Patterns and Esophageal Cancer Risk

**DOI:** 10.7759/cureus.110537

**Published:** 2026-06-09

**Authors:** Rabia Anwar

**Affiliations:** 1 Internal Medicine, Mohi-ud-Din Medical College, Sargodha, PAK

**Keywords:** dietary patterns, epidemiology and biostatistics, heterogeneity, irregular diet, meta-analysis, observational study, oesophageal cancer, publication bias

## Abstract

Irregular dietary patterns have been linked to an increased risk of esophageal cancer in observational meta-analyses. However, methodological limitations may influence the strength and interpretation of these associations. This editorial highlights key concerns, including substantial between-study heterogeneity, inconsistent definitions of dietary irregularity, limited power to assess publication bias, and residual confounding inherent to observational research. Variability in esophageal cancer subtypes and cultural dietary practices further complicates pooled risk estimation. While existing evidence underscores the potential relevance of meal regularity in cancer prevention, cautious interpretation is warranted. Future studies using standardized exposure definitions and subtype-specific analyses are needed to improve the reliability of meta-analytic findings.

## Editorial

Irregular dietary patterns have increasingly been examined as modifiable lifestyle factors associated with cancer risk. A recent meta-analysis by Guan et al. synthesized available observational evidence and reported a strong association between irregular eating habits and esophageal cancer, with a pooled relative risk exceeding fourfold [1]. While these findings draw attention to the potential importance of meal regularity in cancer prevention, several methodological considerations warrant careful interpretation of the reported effect size.

One notable issue is the presence of substantial between-study heterogeneity, with an I² value of 66%, indicating considerable variability across included studies. Although subgroup analyses were performed based on participant characteristics and geographic region, further stratification by esophageal cancer subtype, specifically adenocarcinoma versus squamous cell carcinoma, may have enhanced interpretability [2]. These subtypes differ markedly in their epidemiology, underlying risk factors, and geographic distribution, and pooling them together may obscure important etiologic differences.

Another key limitation relates to the definition and measurement of “irregular diet.” Across observational studies, this exposure is unlikely to be standardized and may encompass heterogeneous behaviors, including inconsistent meal timing, skipping meals, or irregular daily caloric distribution [3]. Variability in dietary assessment methods, reliance on self-reported data, recall bias, and cultural differences in eating patterns may all influence exposure classification and contribute to inflated pooled estimates. The use of validated and uniform criteria for defining dietary irregularity would strengthen future meta-analytic conclusions.

The assessment of publication bias also deserves cautious interpretation. Although formal testing did not demonstrate significant bias, the limited number of included studies--five cohort studies and one case-control study--restricts the statistical power of funnel plot asymmetry and regression-based tests [4]. In lifestyle-related epidemiological research, small-study effects may exaggerate observed associations, particularly when exposure definitions are broad or inconsistently applied.

Finally, the exclusive reliance on observational data limits causal inference. Residual confounding remains a concern, as factors such as tobacco use, alcohol consumption, body mass index, gastroesophageal reflux disease, and overall dietary quality may partially mediate or confound the relationship between irregular eating patterns and esophageal cancer risk. Even with multivariable adjustment, these interrelated exposures are difficult to fully disentangle.

Despite these limitations, the existing evidence highlights an emerging area of interest in nutritional epidemiology and cancer prevention. Future research incorporating standardized exposure definitions, subtype-specific analyses, and more geographically diverse populations will be essential to clarify the true magnitude and clinical relevance of the association between irregular dietary behaviors and esophageal cancer risk.

## References

[1] Guan J, Pan X, Ruan S, He X, Xu Y, Rong X, Ou Y (2022). Relationship between irregular diet and risk of esophageal cancer: a meta-analysis. Front Genet.

[2] Thrift AP, Whiteman DC (2012). The incidence of esophageal adenocarcinoma continues to rise: analysis of period and birth cohort effects on recent trends. Ann Oncol.

[3] Kipnis V, Subar AF, Midthune D (2003). Structure of dietary measurement error: results of the OPEN biomarker study. Am J Epidemiol.

[4] Sterne JA, Sutton AJ, Ioannidis JP (2011). Recommendations for examining and interpreting funnel plot asymmetry in meta-analyses of randomised controlled trials. BMJ.

